# Iron is essential for living!

**DOI:** 10.1186/s13054-014-0678-7

**Published:** 2014-12-08

**Authors:** Sigismond Lasocki, Thomas Gaillard, Emmanuel Rineau

**Affiliations:** Département Anesthésie Réanimation, CHU Angers, 49933, Angers, Cedex 9 France

## Abstract

Iron as an element is a double-edged sword, essential for living but also potentially toxic through the generation of oxidative stress. The recent study by Chen and colleagues in *Critical Care* reminds us of this elegantly. In a mouse model of acute lung injury, they showed that silencing hepcidin (the master regulator of iron metabolism) locally in airway epithelial cells aggravates lung injury by increasing the release of iron from alveolar macrophages, which in turn enhances pulmonary bacterial growth and reduces the macrophages’ killing properties. This work underscores that hepcidin acts not only systematically (as a hormone) but also locally for iron metabolism regulation. This opens areas of research for sepsis treatment but also for iron deficiency or anaemia treatment, since the local and systemic iron regulation appear to be independent.

Iron is essential for living - this is the first lesson from the interesting work from Chen and co-workers published in *Critical Care* [1]. The second and most important lesson is that hepcidin, the master regulator of iron metabolism at the scale of the whole organism [2,3], also plays an important role locally, at the organ level, more specifically in the lung in this study.

Iron is crucial for every kind of living organism, including plants, bacteria, animals and humans, to transport oxygen (through the haemoglobin in animals and humans) and to produce energy (through electron transfer in the mitochondrial respiratory chain). In addition, iron is essential for many metabolic processes, including DNA repair and replication, regulation of gene expression and so on. Many pathogens are thus highly dependent on iron supply and use different pathways to acquire or even steal iron from the environment or from their host [4,5]. These functions of iron are mainly based on its ability to donate electrons, which also allows for the production of free radicals, which are potentially toxic for the host or the invaders. Iron is thus both essential for living and potentially toxic. This is why its metabolism is finely tuned by the hormone hepcidin. Hepcidin was discovered in the early 2000s when searching for new antimicrobial peptides [6,7]. It is now clear that hepcidin plays a key role in the homeostasis of iron: it is synthesized by the liver and acts as a hormone, at different sites (mainly the duodenal enterocytes, where it prevents dietary iron absorption, and the macrophages of the reticulo-endothelial system, where it prevents iron release from stores) [2,3]. Hepcidin acts through binding to ferroportin, the sole known iron exporter [8].

Besides its role in iron metabolism regulation, hepcidin may also have a role locally, as pointed out by Chen and colleagues in their work [1]. They used an elegant mouse model to silence hepcidin expression in airway epithelial cells. This knockdown of hepcidin induced a decrease in hepcidin expression (at the RNA and protein levels) in these cells and higher ferroportin and lower iron content in alveolar macrophages. This indicates that hepcidin produced by the airway epithelial cells acts locally on alveolar macrophages as a paracrine hormone. Indeed, no difference was observed in serum iron or in spleen iron stores, indicating no modification of systemic iron. Using a model of acute lung injury secondary to peritonitis, Chen and colleagues show that the knockdown of hepcidin aggravates the acute lung injury (with increased mortality) probably through two main factors: an exacerbation of lung sepsis, thanks to an impaired phagocytic activity of alveolar macrophages (the reduced iron content of macrophages may prevent their ability to produce oxidative stress) and possibly thanks to an increase of iron availability for pathogens leading to higher bacterial load in the lungs [9]; and an increase in alveolar damage due to iron toxicity secondary to its release from macrophages (but this remains hypothetical).

The increase in pulmonary bacterial colonization in the hepcidin knockdown mice may also indicate that hepcidin has a direct antimicrobial effect. Indeed, hepcidin is a member of the β-defensin peptide family. Defensins are small peptides, rich in cysteine and active in host defence against bacteria but also against some viruses and fungi. They are produced by cells of the immune system and act by binding to the pathogen membrane to form pores that induce the killing of the cell. They play an important role in immunity in the lung [10].

This work underscores the role of hepcidin in lung protection against pathogens and may also help in understanding that iron metabolism regulation is compartmentalized. Locally, in this case in the lung, the organism prevents iron release and produces hepcidin to help fight invaders [11]. But iron is needed for erythropoiesis and systemic iron infusion may be useful and not deleterious, even in the presence of sepsis, thanks to regulation of hepcidin synthesis at the systemic level [12]. This separated regulation could allow iron treatment of critically ill patients, even in the presence of inflammation and or sepsis [13].

In the future, regulation of iron metabolism through hepcidin manipulation could be carried out at two levels: locally to enhance host defence (through hepcidin synthesis induction), and systemically to prevent anaemia of inflammation (through hepcidin inhibition). Iron and hepcidin are really essential for living!
